# Hierarchical poly(2-aminothiophenol)/Co–Ni heterostructures with nanoflake–nanoneedle architecture for high-performance supercapacitors

**DOI:** 10.1039/d5ra09024e

**Published:** 2026-05-08

**Authors:** Aya Fathy, Ashour M. Ahmed, M. A. Basyooni-M. Kabatas, Mamduh J. Aljaafreh, Mohamed Shaban, Hany Hamdy, Mohamed Rabia

**Affiliations:** a Nanophotonics and Applications Lab, Physics Department, Faculty of Science, Beni-Suef University Beni-Suef 62514 Egypt; b Physics Department, College of Science, Imam Mohammad Ibn Saud Islamic University (IMSIU) Riyadh 11623 Saudi Arabia; c Department of Precision and Microsystems Engineering, Delft University of Technology Mekelweg 2, 2628 CD Delft The Netherlands m.kabatas@tudelft.nl; d Institute of Nanotechnology (INT), Karlsruhe Institute of Technology (KIT) Kaiserstraße 12 76131 Karlsruhe Germany m.kabatas@kit.edu; e Physics Department, Faculty of Science, Islamic University of Madinah Madinah Saudi Arabia; f Nanomaterials Science Research Laboratory, Chemistry Department, Faculty of Science, Beni-Suef University Beni-Suef Egypt

## Abstract

The present study reports a hierarchical supercapacitor electrode that integrates poly(2-aminothiophenol) (P2-ATH) with a cobalt–nickel heterostructure comprising cobalt carbonate hydroxide hydrate (CCHH) and cobalt-nickel oxide (CNO). The hybrid is synthesized by hydrothermal growth of CCHH/CNO nanoneedles, followed by *in situ* oxidative polymerization of P2-ATH to yield conformal nanoflakes. This interpenetrating architecture furnishes a porous, electrically percolated network that shortens ion-diffusion paths and accelerates electron transport, thereby coupling the redox activity of P2-ATH with the multiple Faradaic sites of the Co–Ni phase. Electrochemical tests in different electrolytes (NaOH, NaCl, and HCl) demonstrate a strong electrolyte dependence, with 0.5 M HCl yielding the best performance. At 0.4 A g^−1^, the specific capacitance reaches 113.87 F g^−1^ in HCl, compared with 27.89 F g^−1^ in NaOH and 7.73 F g^−1^ in NaCl. In 0.5 M HCl, the electrode delivers an energy density of 5.69 Wh kg^−1^ at a specific power of 479.7 W kg^−1^. The results highlight the synergistic interplay between the conductive P2-ATH and the Co–Ni nanoneedle, establishing P2-ATH/CNO-CCHH as a promising platform for high-rate, durable supercapacitors and broader electrochemical energy-storage applications.

## Abstract 2

Hierarchical P2‑ATH nanoflakes grown on Co–Ni nanoneedles (CCHH/CNO) form an interpenetrating porous network for rapid ion and electron transport.

## Graphical abstract

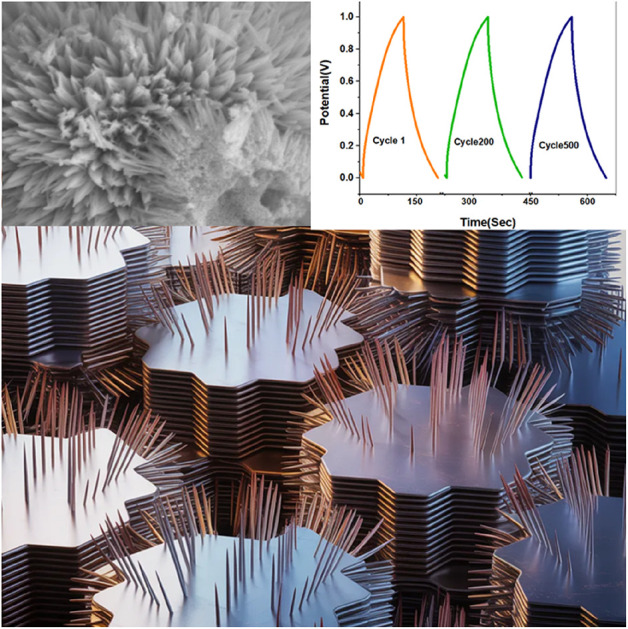

## 1 Introduction

The growing global energy demand and increasing environmental concerns necessitate advancing renewable energy technologies and developing more effective energy storage systems.^1,2^ Electrochemical supercapacitors (SCs) have emerged as promising devices for storing electric energy generated from renewable sources.^3–5^ SCs have attracted considerable attention because of their simple fabrication, exceptional power density, and long-term cycling stability.^6^ Unlike traditional capacitors, supercapacitors offer significantly higher energy and power densities, enabling rapid charge–discharge processes critical for modern technological applications. Supercapacitors are classified into pseudocapacitors and electric double-layer capacitors (EDLCs).^7,8^ Pseudocapacitors rely on rapid, reversible Faradaic redox reactions, whereas EDLCs store energy by physically adsorbing and desorbing ions at the electrode/electrolyte interface. Pseudocapacitors are advantageous for high-power and energy density applications.^9^

The characteristics of its electrode materials strongly influence a supercapacitor's performance. Electrode architectures with high electrochemical redox activity are essential for efficient energy storage. Recently, a wide range of carbonate hydroxides has been investigated for electrochemical energy storage applications. Among them, cobalt carbonate hydroxide hydrate (CCHH) has attracted considerable attention due to its multiple oxidation states and unique morphology, which facilitate efficient redox reactions and enhance charge transport. The existence of hydrophilic CO_3_^2−^ ions improves electrolyte wettability. Moreover, its layered structure and rich redox activity enable facile ion diffusion and mass transfer at the electrode–electrolyte interface.^10^ Additionally, CCHH offers a high surface area and a large three-dimensional (3D) framework that can be synthesized through straightforward processes.^11^ Despite these advantages, the application of CCHH in supercapacitors remains limited due to its intrinsically low electrical conductivity.^12^ To overcome this drawback, a promising strategy is to combine CCHH with highly conductive materials, facilitating electron transport and enhancing electrochemical properties.

Nickel-based electrode materials have been studied for their high theoretical specific capacity, while cobalt is often used to control morphology and improve conductivity. Both cobalt- and nickel-based compounds are attractive due to their environmental compatibility, multiple oxidation states, cost-effectiveness, and excellent redox activity.^13,14^ However, their practical supercapacitor applications are limited by low rate capability and limited cycling stability.^15^ To address these limitations, bimetallic nickel/cobalt (Ni/Co) systems have been explored for their synergistic effects, which improve both chemical stability and redox activity. In particular, CNO, Co_1.29_Ni_1.71_O_4_, exhibits enhanced electrical conductivity due to the cooperative behavior of the two metal centers.^10^ The complementary characteristics of cobalt and nickel in bimetallic systems provide superior cycling stability and improve electrochemical behavior compared to their monometallic counterparts.^16^

In addition, conducting polymers (CPs) are widely recognized as suitable materials for supercapacitor electrodes.^17,18^ CPs offer several advantages, including high electrical conductivity, good energy storage capability, low density, and low production cost.^19^ When structured at the nanoscale, CPs contribute to supercapacitor electrodes with enhanced mechanical stability, elevated power density, and superior cycling durability.

On the other side, poly(2-aminothiophenol) (P2-ATH; C_6_H_7_NS) is a fascinating material due to its unique bifunctional nature, which distinguishes it from aniline and other substituted anilines. P2-ATH contains both amine (–NH_2_) and thiol (–SH) groups, providing multiple reactive sites that enhance its chemical versatility. The monomer 2-aminothiophenol (ATP) has attracted attention for the fabrication of three-dimensional (3D) and two-dimensional (2D) nanoparticle assemblies *via* electrostatic interactions or covalent bonding. The differing reactivity of the thiol and amine functional groups in ATP has been efficiently exploited to engineer molecular assemblies with tailored morphologies and tunable surface properties.^20,21^ Additionally, the presence of the phenyl ring in ATP enhances electrical coupling between the electrode surface and attached nanoparticles, further improving the performance of the resulting material. Coating nanoparticles with P2-ATH provides them with a high affinity for metal ions, owing to the strong coordination capabilities of nitrogen and sulfur atoms and the π-electrons in the aromatic ring structure of the repeating units.^22,23^ These features make P2-ATH a promising candidate for advanced applications, including light-emitting diodes (LEDs), energy storage devices, and sensors.^24^

Incorporating conductive polymers (CPs) with bimetallic inorganic materials such as metal oxides into heterostructure electrodes is a highly effective strategy to exploit their complementary properties through synergistic interactions.^25^ Conductive polymers provide a flexible, tunable, and electrically conductive framework, with conjugated π-electron systems that facilitate rapid electron transport and redox activity. Meanwhile, bimetallic inorganic components offer excellent electrochemical and structural stability, as well as multiple reversible redox sites. These result in significantly increased capacitance and improved energy and power densities compared to either component alone. Moreover, CPs help buffer volume changes and mechanical stress in metal oxides during charge–discharge cycles, thus enhancing cycling stability and reducing material degradation. Redox pseudocapacitance from CPs (*via* doping/dedoping mechanisms) and surface- or intercalation-based capacitance from the bimetallic inorganic phase (*via* metal–ion redox transitions) work in tandem to improve overall performance. Direct, intimate interfacial bonding between the polymer and inorganic phases lowers charge-transfer resistance and accelerates electrochemical kinetics. Additionally, the formation of hierarchical nanostructures increases surface area, promotes ion accessibility, and enhances mechanical integrity. As a result, CP-inorganic heterostructures are considered promising electrode materials for generating supercapacitors.

In this study, a novel nanostructured electrode composed of poly(2-aminothiophenol) (P2-ATH) integrated with CNO-CCHH was prepared for the first time using a combination of hydrothermal synthesis and *in situ* oxidative polymerization. The resulting P2-ATH/CNO-CCHH heterostructure features a unique nanoflake-nanoneedle architecture, characterized by a high surface area, enhanced electrical conductivity, and efficient electron transport pathways. The synergistic interaction between the conductive polymer and the bimetallic inorganic phases significantly enhances charge storage capacity and stability. The electrode's performance was assessed using galvanostatic charge–discharge (GCD) and cyclic voltammetry (CV) in various electrolytes, including NaOH, NaCl, and HCl. Notably, at a current density of 0.4 A g^−1^ in 0.5 M HCl, the heterostructure achieved a high specific capacitance of 113.87 F g^−1^, along with power and energy densities of 479.7 W kg^−1^ and 5.69 Wh kg^−1^, respectively. These results demonstrate the strong potential of the P2-ATH/CNO-CCHH heterostructure as a novel electrode material for high-performance supercapacitor applications.

## 2 Experimental steps

### 2.1 Chemicals and materials

Ammonium persulfate [(NH_4_)_2_S_2_O_8_, 98%], nickel nitrate hexahydrate [Ni(NO_3_)_2_)·6H_2_O, 99%], urea [NH_2_CONH_2_, 99%], cobalt nitrate hexahydrate [Co(NO_3_)_2_)·6H_2_O, 98%] were acquired from Al-Nasr Company, Egypt. Nafion solution (5 wt%) was obtained from Sigma-Aldrich, USA. Loba Chemie, India, supplied sulfuric acid [H_2_SO_4_, 95%] and acetic acid [CH_3_COOH, 99%]. 2-Aminothiophenol monomer was purchased from ALPHA CHEMIKA, India. Deionized (DI) water was used throughout all experimental procedures to prevent contamination from dissolved ions.

### 2.2 Preparation of P2-ATH/CNO-CCHH heterostructure

The P2-ATH/CNO-CCHH heterostructure was synthesized using a two-step hydrothermal synthesis process followed by *in situ* oxidative polymerization, as shown schematically in Fig. S1. In the first step, CNO-CCHH nanopowder was prepared *via* a hydrothermal method. Specifically, 2.0 g of cobalt nitrate hexahydrate and 2.0 g of nickel nitrate hexahydrate were dissolved in 70.0 mL of DI water. Subsequently, 2.20 g of urea was introduced into the precursor solution, followed by continuous stirring for 0.5 h. The prepared solution was placed into a 100 mL stainless-steel autoclave lined with Teflon and subjected to hydrothermal treatment at 120 °C for 9 hours. The obtained precipitate was thoroughly washed several times with distilled ethanol and water. The sample was dried at 60 °C for 6 hours, followed by calcination at 400 °C for 3 hours, yielding the final CNO-CCHH nanopowder.

In the second step, the P2-ATH/CNO-CCHH heterostructure was synthesized *via in situ* oxidative polymerization of poly(2-aminothiophenol) onto the CNO-CCHH surface. About 0.2 g of the prepared CNO-CCHH nanopowder was dispersed in a 0.01 M solution of 2-aminothiophenol monomer under constant stirring. Then, 0.15 M ammonium persulfate solution was slowly added as the oxidizing agent. The reaction mixture was stirred magnetically for 2 hours, leading to the polymerization of P2-ATH on the surface of the CNO-CCHH nanopowder. The resulting P2-ATH/CNO-CCHH heterostructure was filtered through Whatman filter paper and washed several times with water. The final product was oven-dried at 80 °C for 12 hours, then ground meticulously with an agate mortar to produce a uniform powder.

### 2.3 Analytical techniques

The crystal structure and phase composition of the P2-ATH/CNO-CCHH heterostructure were analyzed using X-ray diffraction (XRD) with a PANalytical instrument. The XRD measurements were performed with Cu Kα radiation (wavelength = 0.154 nm) at 15 mA and 35 kV. Data were collected with a step size of 0.05° in the 2*θ* range of 5° to 80°. The resulting diffraction patterns were investigated using X'Pert HighScore software (Malvern Panalytical Ltd). The microstructure and morphology of the heterostructure were examined using transmission electron microscopy (ZEISS EVOMA10) and scanning electron microscopy (JEOL JSM5410LV). The elemental composition was determined using an energy-dispersive X-ray spectroscopy (EDX) unit attached to an SEM device. Fourier-transform infrared spectroscopy (FTIR-8400S; Shimadzu) was employed to identify the functional groups in the heterostructure. The FTIR measurements were conducted at an incident angle of 50°, using KBr pellets prepared by compressing a mixture of 5 wt% of the heterostructure and 95 wt% % KBr under a hydraulic press. The elemental composition and oxidation states within the heterostructure were further investigated using X-ray photoelectron spectroscopy (XPS, Thermo Scientific K-Alpha).

### 2.4 Electrode fabrication

To prepare the working electrode, 0.04 g of the P2-ATH/CNO-CCHH heterostructure was dispersed in a mixture containing 100 µL of Nafion and 0.75 mL of ethanol. The suspension was continuously stirred for 13 hours to get a well-dispersed slurry. Subsequently, 50 µL of the slurry was drop-cast onto a 1 cm^2^ gold (Au) sheet and allowed to dry at room temperature, forming the active electrode layer. A symmetric supercapacitor cell was fabricated by placing two identical P2-ATH/CNO-CCHH/Au electrodes in a face-to-face configuration. Electrode separation was accomplished by inserting a filter paper between the two electrodes. Before assembly, the filter paper was soaked overnight in a 0.5 M electrolyte solution of NaOH, NaCl, or HCl. This pre-soaked separator facilitated efficient ionic conduction across the electrode interface while reducing internal resistance during electrochemical testing.

Gold was selected as the current collector because of its excellent electrical conductivity, high chemical stability, and electrochemical inertness over the investigated potential range. In acidic, neutral, and alkaline electrolytes, Au is highly resistant to corrosion, oxidation, and surface passivation, thereby minimizing parasitic side reactions and ensuring that the measured electrochemical response arises predominantly from the active P2-ATH/CNO-CCHH heterostructure rather than from the substrate. In addition, the smooth and conductive Au surface promotes uniform slurry deposition, strong adhesion of the active material, and efficient electron transport across the electrode/current collector interface. These characteristics improve charge-transfer efficiency and enhance the reliability and reproducibility of the electrochemical measurements.

### 2.5 Electrochemical characterizations

The electrochemical performance of the P2-ATH/CNO-CCHH electrode for supercapacitor applications was evaluated using galvanostatic charge–discharge (GCD) and cyclic voltammetry (CV) measurements. All measurements were performed in a symmetric two-electrode configuration using a workstation (CHI 660E, CH Instruments) at 25 °C. Electrochemical tests were conducted in three electrolytes (0.5 M NaCl, 0.5 M NaOH, and 0.5 M HCl). CV was conducted over a potential window of 0.0–1.0 V at various scan rates, while GCD measurements were performed at different current densities.

## 3 Results and discussion

### 3.1 Characterization of the nanostructures

#### 3.1.1 XRD analysis

**Fig. 1:**
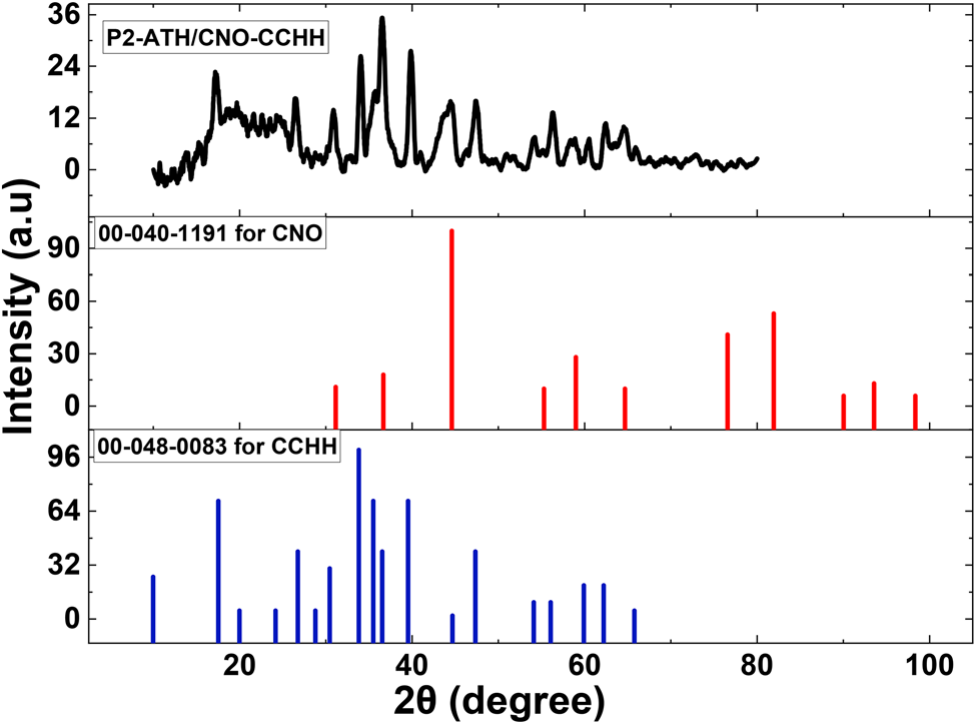
The XRD spectrum for P2-ATH/CNO-CCHH heterostructure with standard XRD patterns for CCHH and CNO.

Fig. 1 shows the XRD pattern of the fabricated P2-ATH/CNO-CCHH heterostructure, alongside the standard reference patterns for CCHH and CNO. The broad diffraction peak centered around 2*θ* ≈ 23° is attributed to the periodic alignment of polymer chains in P2-ATH, which is characteristic of its amorphous nature.^22,26,27^ Such broad features are commonly observed in polymers due to chain disorder, branching, or structural heterogeneity. This amorphous nature may offer more accessible active sites for ion transport. The diffraction peaks at 2*θ* values of 26.67°, 34.05°, 39.91°, 47.43°, 54.05°, 56.30°, and 62.41° are indexed to the (220), (221), (231), (340), (060), (142), and (450) planes, respectively. These peaks correspond to cobalt carbonate hydroxide [Co(CO_3_)_0.5_(OH)·0.11 H_2_O], which crystallizes in an orthorhombic structure consistent with the standard pattern reported in JCPDS card no. 00-048-0083.

The formation of carbonate and hydroxide phases is likely due to the thermal decomposition of urea, which provides both ions in the reaction medium. Such mixed-metal hydroxide-based structures provide abundant redox-active sites, which are beneficial for pseudocapacitive behavior.

In addition, peaks at 2*θ* values of 30.87°, 36.63°, 44.67°, and 64.51° are attributed to the (220), (311), (400), and (440) planes of the cubic phase of cobalt-nickel oxide [Co_1.29_Ni_1.71_O_4_], in accordance with JCPDS card no. 00-040-1191. This indicates successful formation of the bimetallic oxide through cation substitution between Co and Ni ions. This structure typically exhibits excellent electrical conductivity and structural stability, making it highly suitable for electrochemical applications.

The average crystallite sizes of the individual components, calculated using the Scherrer equation, were approximately 9.28 nm for P2-ATH, 20.59 nm for CCHH, and 16.14 nm for CNO. These nanoscale dimensions confirm the nanocrystalline nature of the synthesized heterostructure, providing a larger surface area, shorter ion diffusion paths, and more active sites for redox reactions. Hence, the XRD analysis confirms the successful incorporation of amorphous P2-ATH into a hybrid heterostructure with crystalline CCHH and CNO phases.

#### 3.1.2 FTIR analysis

**Fig. 2:**
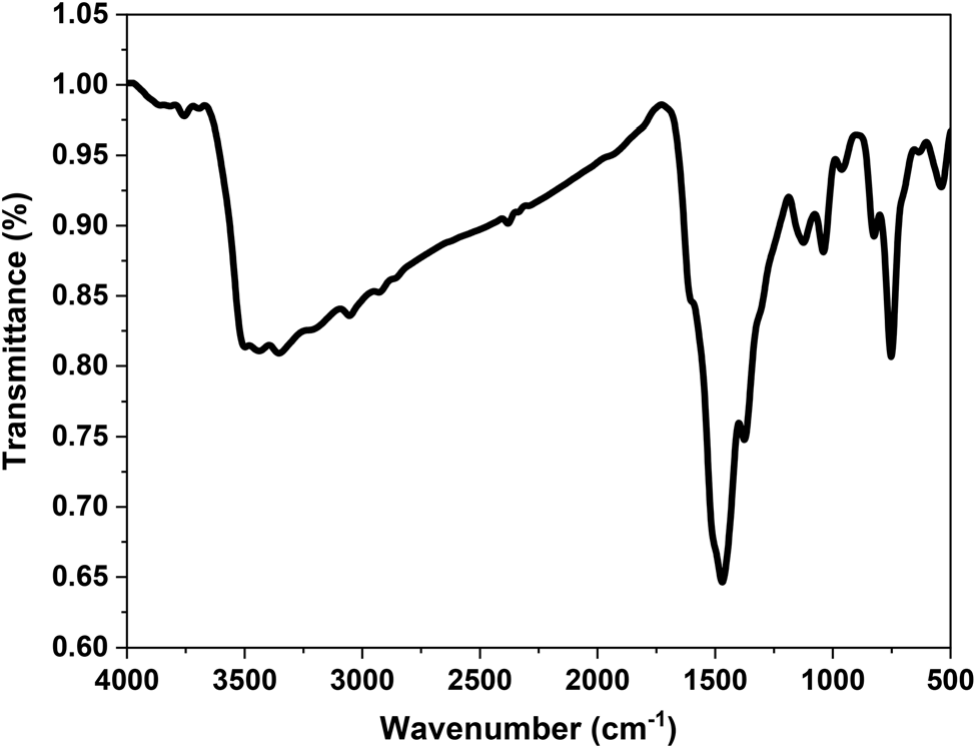
FTIR spectra of P2-ATH/CNO-CCHH heterostructure.

Fig. 2 presents the FTIR spectrum of the synthesized P2-ATH/CNO-CCHH heterostructure. Table S1 lists the characteristic functional groups of P2-ATH and the metal components [S1–S13]. The broad absorption band detected in the 3354 to 3438.1 cm^−1^ range corresponds to the stretching vibrations of N–H and S–H bonds, confirming the presence of the primary amine group in the P2-ATH polymer.^28,29^ These bonds serve as essential coordination sites for binding with metal ions and enhancing interfacial charge transfer. The band at 3054.4 cm^−1^ is attributed to the C–H stretching vibrations of various alkane groups.^30^ The bands at 1470.1 cm^−1^ correspond to C=C stretching vibrations in the benzenoid ring and C–N stretching.^31,32^

Notably, shifts in the positions of the C=C, C–H, C–N, and N–H peaks are observed, indicating chemical interaction between the P2-ATH matrix and the incorporated CNO-CCHH components. These shifts suggest the successful incorporation of the polymer backbone's bimetallic oxide/carbonate phase. The band at 634.4 cm^−1^ is indexed to carbonate group vibrations,^33,34^ consistent with the formation of cobalt carbonate hydroxide hydrate (CCHH), as previously confirmed by XRD. Strong bands corresponding to metal–oxygen and metal–hydroxide vibrations appear in the lower wavenumber region. The peak at 539.2 cm^−1^ is attributed to overlapping Co–O and Ni–O bond vibrations,^35^ providing direct evidence of the coexistence of cobalt and nickel oxides in the heterostructure. This confirms the successful formation of the bimetallic cobalt-nickel oxide (CNO) structure. Additionally, the band detected at 451.5 cm^−1^ corresponds to the Co–OH stretching vibration,^34^ indicating the presence of cobalt hydroxide species in the final heterostructure.

#### 3.1.3 SEM analysis

**Fig. 3:**
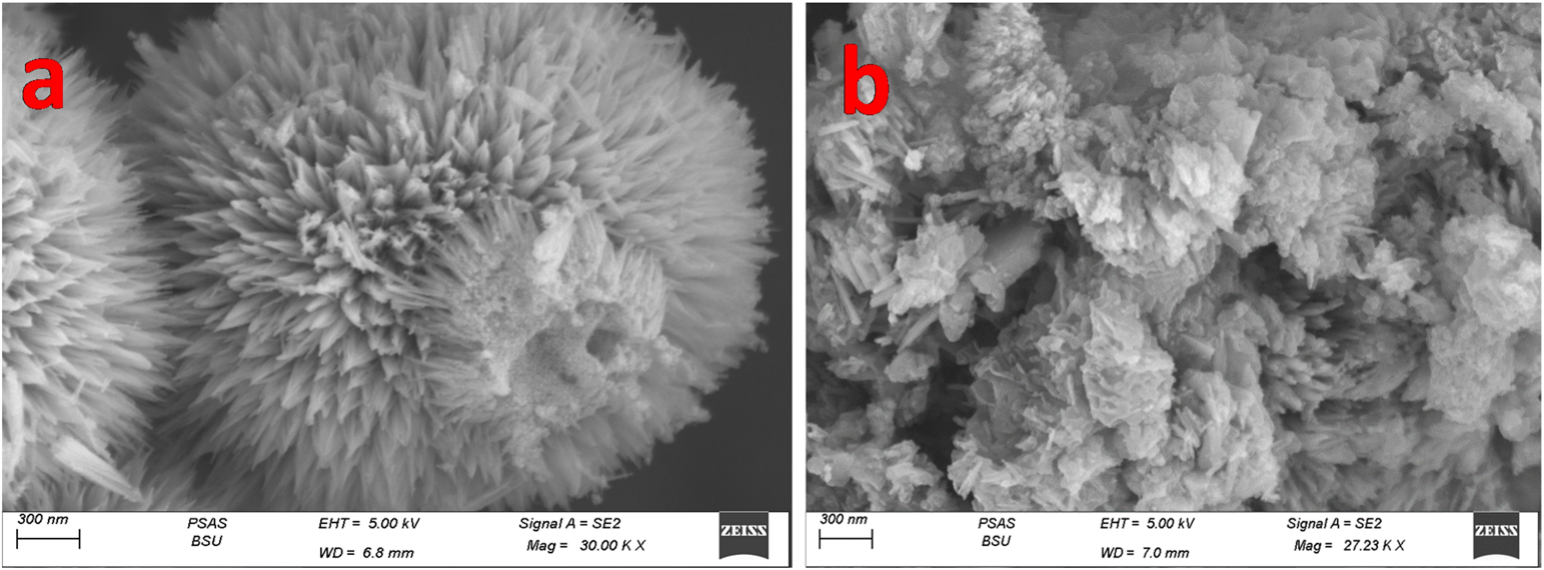
SEM images of (a) CNO-CCHH and (b) P2-ATH/CNO-CCHH.

Fig. 3 presents SEM images of the morphology of pristine CNO-CCHH and the P2-ATH/CNO-CCHH heterostructure. In Fig. 3(a), the CNO-CCHH sample exhibits a distinct nanoneedle morphology, with elongated needles radiating outward from central nucleation sites, forming spherical, flower-like clusters. These nanoneedles are relatively uniform, with lengths extending to several hundred nanometers and diameters in the tens of nanometers range. Such a structure provides abundant active sites, high specific surface area, and short ion diffusion paths, all of which are favorable for electrochemical energy storage. The ordered and crystalline arrangement of the nanoneedles further contributes to structural stability and enhanced conductivity.

The morphology resulting from the *in situ* oxidative polymerization of P2-ATH onto the CNO-CCHH nanoneedles is illustrated in Fig. 3(b). While the needle-like features are still visible, they appear partially coated or embedded in flaky domains, which is characteristic of polymer nanoflakes. This indicates successful deposition of the P2-ATH polymer matrix, which forms a conformal coating on the nanoneedles. The polymer nanoflakes modify the surface texture, enhance conductivity, and introduce redox-active functional groups. The coexistence of nanoflakes and nanoneedles yields a hierarchical heterostructure that combines a high surface area with efficient charge transport pathways. This architecture enhances mechanical stability, promotes rapid ion and electron transfer, and strengthens interfacial contact between the polymer and inorganic components. Such intimate interfacial bonding improves electrochemical performance and ensures long-term cycling stability while minimizing internal resistance.

#### 3.1.4 EDX analysis

**Fig. 4:**
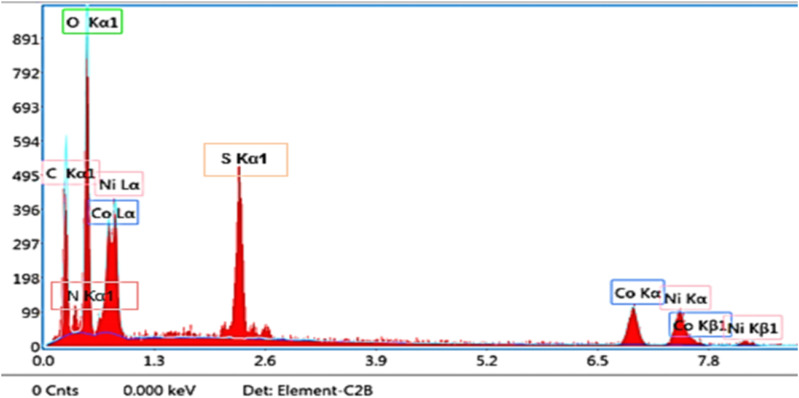
EDX elemental analysis of P2-ATH/CNO-CHH.

The EDX spectrum of the P2-ATH/CNO-CCHH heterostructure is shown in Fig. 4. The detected peaks correspond to the characteristic X-ray energies of the constituent elements excited by the electron beam. Strong peaks are observed for oxygen (O Kα1), carbon (C Kα1), nickel (Ni Lα and Ni Kα/Kβ), and cobalt (Co Lα, Co Kα/Kβ), along with a distinct sulfur (S Kα1) peak. The well-resolved elemental peaks and the absence of extraneous impurity signals confirm the heterostructure's high purity and successful synthesis.

Quantitative analysis reveals the elemental compositions to be C (29.4%), O (22.3%), S (5.6%), N (11.2%), Co (14.7%), and Ni (15.8%). The Co Kα and Kβ peaks are located at 6.924 keV and 7.85 keV, respectively, while the Co Lα peak is detected at 0.776 keV. Similarly, the Ni Kα peak appears near 7.5 keV with corresponding Kβ peaks, confirming the presence of nickel in both L and K series. The relative atomic ratio suggests a nickel-rich bimetallic phase, consistent with the formation of cobalt-nickel oxide (CNO). The pronounced oxygen peak further supports the coexistence of oxidized metal species, consistent with the expected formation of CNO and cobalt carbonate hydroxide hydrate (CCHH) phases. Meanwhile, the carbon peak is attributed mainly to the P2-ATH polymer backbone, which contains aromatic rings and alkyl groups.

#### 3.1.5 TEM analysis

**Fig. 5:**
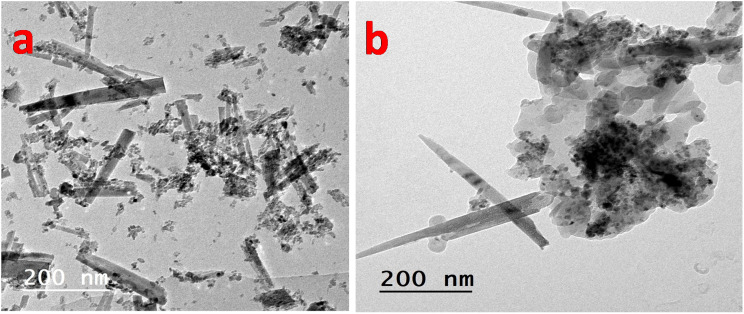
TEM images of (a) CNO-CCHH nanoneedles and (b) P2-ATH/CNO-CCHH heterostructure.

Fig. 5 presents TEM images that elucidate both the morphological evolution and the interfacial architecture of the P2-ATH/CNO-CCHH system. As shown in Fig. 5(a), the pristine CNO-CCHH material is composed of elongated nanoneedles with a high aspect ratio and well-defined crystalline features. The sharp edges and uniform contrast indicate a high degree of crystallinity. These nanoneedles are arranged in an intertwined, open-framework network that generates a mechanically robust three-dimensional architecture. Such a configuration provides interconnected ion diffusion pathways and facilitates electrolyte penetration into the internal structure, which is essential for high-rate electrochemical performance.

Following *in situ* polymerization, Fig. 5(b) reveals the formation of a hierarchically integrated heterostructure with a markedly different morphology. The P2-ATH polymer appears as a semi-amorphous, nanoflake-like coating that conformally encapsulates and partially embeds the nanoneedles. Despite this modification, the one-dimensional framework of the CNO-CCHH phase remains preserved, indicating that the polymerization process does not disrupt the crystalline backbone. Instead, the system evolves into a dual-scale architecture in which crystalline nanoneedles coexist with amorphous polymer domains, leading to increased surface roughness and structural complexity.

At the interfacial level, TEM clearly demonstrates an intimate and coherent contact between the polymer and oxide phases, indicating strong interfacial adhesion. This suggests that P2-ATH is not merely physically deposited but is chemically anchored and interpenetrated within the oxide scaffold. Such interactions are likely mediated by coordination bonding between the metal ions (Ni and Co) and the functional groups of the polymer (–NH_2_ and –SH), resulting in effective electronic coupling across the interface. The coexistence of crystalline inorganic domains and amorphous polymeric regions thus establishes a hierarchical interfacial architecture with complementary functionalities. The nanoneedle framework provides a high-conductivity backbone, whereas the polymer coating enhances surface reactivity and interfacial kinetics. This synergistic integration minimizes charge-transfer resistance, improves ion/electron transport, and ensures efficient utilization of active materials.

#### 3.1.6 XPS analysis

**Fig. 6:**
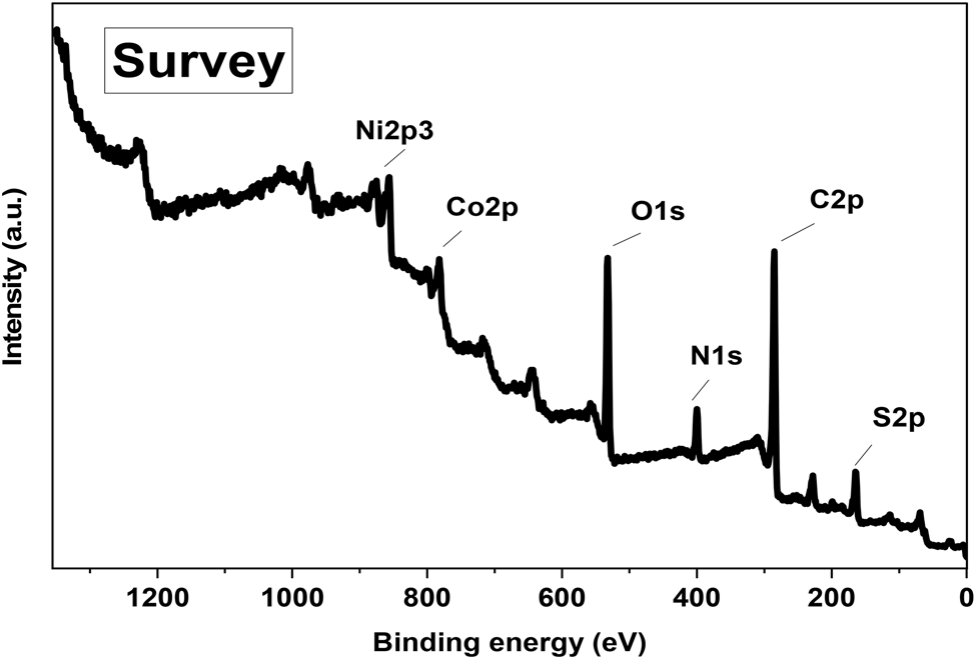
Full survey XPS analysis of P2-ATH/CNO-CCHH heterostructure.

Fig. 6 displays the XPS full-scan spectrum of the heterostructure, highlighting the presence of cobalt, oxygen, nickel, sulfur, carbon, and nitrogen, thereby confirming the successful incorporation of both inorganic and organic components. This broad scan offers comprehensive insight into the elemental distribution within the material. Fig. S2 present high-resolution core-level XPS spectra for Ni 2p, Co 2p, O 1s, N 1s, S 2p, and C 1s. The high-resolution Ni 2p spectrum (Fig. S2a) was deconvoluted into two main spin–orbit doublets corresponding to Ni^2+^ and Ni^3+^ oxidation states. Peaks at 855.6 eV and 873.2 eV are assigned to Ni^2+^, while those at 858.2 eV and 875.7 eV correspond to Ni^3+^. Moreover, the spectrum exhibits four satellite peaks, indicative of strong electron interactions and mixed oxidation states. The splitting and satellite features confirm the coexistence of Ni^2+^/Ni^3+^ species, characteristic of nickel-based oxides involved in redox activity.^36,37^ The Co 2p spectrum is deconvoluted into two spin–orbit doublets corresponding to the Co2+ and Co3+ oxidation states, and four shake-up satellites (Fig. S2b). The peaks observed at 779.6 eV and 796.4 eV are characteristic of Co^3+^, whereas those at 781.2 eV and 799.7 eV correspond to Co^2+^.^38,39^ These findings indicate the coexistence of both oxidation states within the CNO-CCHH material.

The high-resolution N 1s spectrum displays two peaks at 398.7 eV and 399.6 eV, corresponding to N–C and N–H bonds (Fig. S2c). These are attributed to amine functionalities derived from the P2-ATH polymer and confirm the presence of nitrogen-containing active sites in the heterostructure. The C 1s spectrum (Fig. S2d) is deconvoluted into three primary peaks. The binding energy peak at 284.3 eV corresponds to C–C bonds, whereas the peaks at 287.7 eV and 285.6 eV indicate C=C and C–H functional groups, respectively.^40^ The O 1s spectrum (Fig. S2e) reveals many distinct peaks. The peak at 529.0 eV is assigned to lattice oxygen bonded to metal atoms (metal–oxygen bonds), while the peak at 533.3 eV corresponds to surface hydroxyl groups.^41^ A third peak at 531.4 eV is associated with oxygen atoms adjacent to oxygen vacancies, which are known to enhance electrochemical activity in metal oxides and hydroxides.^42^ Finally, the high-resolution S 2p spectrum (Fig. S2f) exhibits two characteristic sulfur-related components centered at 164.5 and 168.2 eV, corresponding to the deconvoluted S 2p_1/2_ and S 2p_3/2_ spin–orbit states.^43–45^ The dominant peak at 164.5 eV is assigned to sulfur in C–S–C/C–S–H environments, corresponding to thiophene-derived sulfur and residual thiol (–SH) functionalities in the P2-ATH backbone. The higher binding energy component at 168.2 eV is attributed to oxidized sulfur species, such as sulfoxide, sulfone, or sulfate-like S–O_*x*_ groups, formed during oxidative polymerization or slight surface oxidation upon air exposure. The relatively low intensity of this peak indicates that sulfur oxidation is limited and does not significantly compromise the structural integrity of P2-ATH. These function groups can further enhance surface polarity, electrolyte wettability, ion accessibility, and interfacial electronic interactions with Co/Ni active centers, thereby improving the overall electrochemical performance of the heterostructure.

#### 3.1.7 BET analysis

**Fig. 7:**
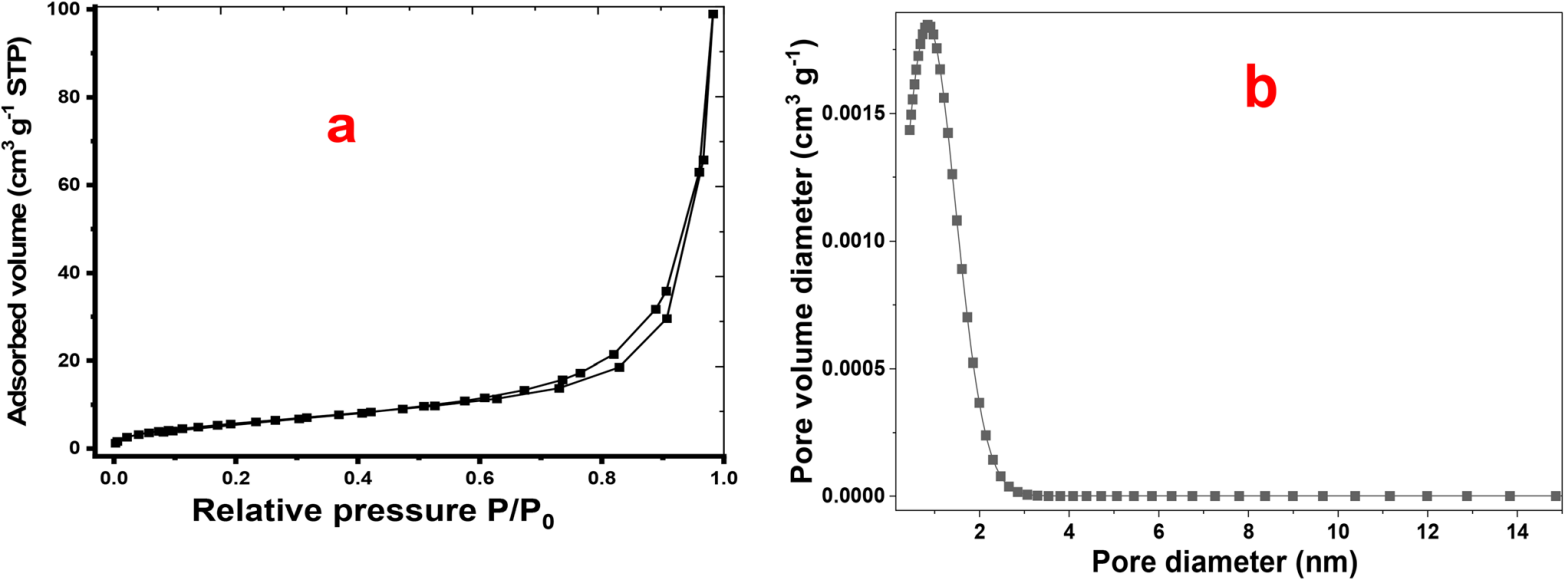
(a) N_2_ adsorption–desorption isotherms and (b) pore size distributions of P2-ATH/CNO-CCHH.

The textural properties of the P2-ATH/CNO-CCHH heterostructure were comprehensively analyzed using Brunauer–Emmett–Teller (BET), Barrett–Joyner–Halenda (BJH), and non-local density functional theory (NLDFT) methods. Fig. 7(a) presents the N_2_ adsorption–desorption isotherms and pore size distributions of the P2-ATH/CNO-CCHH sample based on the physisorption of ultra-high-purity nitrogen gas using a Quantachrome NOVAtouch instrument. The N_2_ adsorption–desorption isotherm exhibits a type IV profile with a distinct H3 hysteresis loop, characteristic of mesoporous materials with slit-shaped pores formed by layered or aggregated nanostructures. This confirms that the integration of P2-ATH does not collapse the porous framework of the CNO-CCHH matrix; rather, it stabilizes the structure and suppresses agglomeration. The absence of a clear adsorption plateau at high relative pressures further indicates the presence of macropores and interparticle voids, suggesting an open, interconnected network that facilitates electrolyte accessibility.

Quantitative BET analysis reveals a specific surface area of approximately 23.37 m^2^ g^−1^, a total pore volume of 0.153 cm^3^ g^−1^, and an average pore diameter of 26.2 nm. The specific surface area arises from the synergistic combination of CNO-CCHH nanoneedles and P2-ATH nanoflakes, providing abundant active sites for faradaic reactions. Moreover, the relatively high pore volume and large pore diameter ensure efficient electrolyte infiltration and reduced ion transport resistance. The gradual adsorption at low relative pressures also suggests the coexistence of micropores, which contribute additional active sites for charge storage.

The BJH pore-size distribution further supports the mesoporous nature of the heterostructure, which facilitates rapid ion diffusion and provides short transport pathways. In parallel, NLDFT analysis reveals a dominant, narrow peak centered at 1.6 nm, as shown in Fig. 7(b). This narrow distribution indicates a relatively uniform pore architecture. Hence, the combined analyses demonstrate that the P2-ATH/CNO-CCHH heterostructure possesses hierarchical porosity and favorable surface characteristics, making it highly suitable for advanced supercapacitor applications.

### 3.2 Electrochemical performance

The electrochemical activity of the P2-ATH/CNO-CCHH heterostructure as a supercapacitor electrode was examined using CV and GCD. At room temperature, studies were performed in 0.5 M NaOH, 0.5 M HCl, and 0.5 M NaCl electrolytes.

#### 3.2.1 Cyclic voltammetry (CV)

**Fig. 8:**
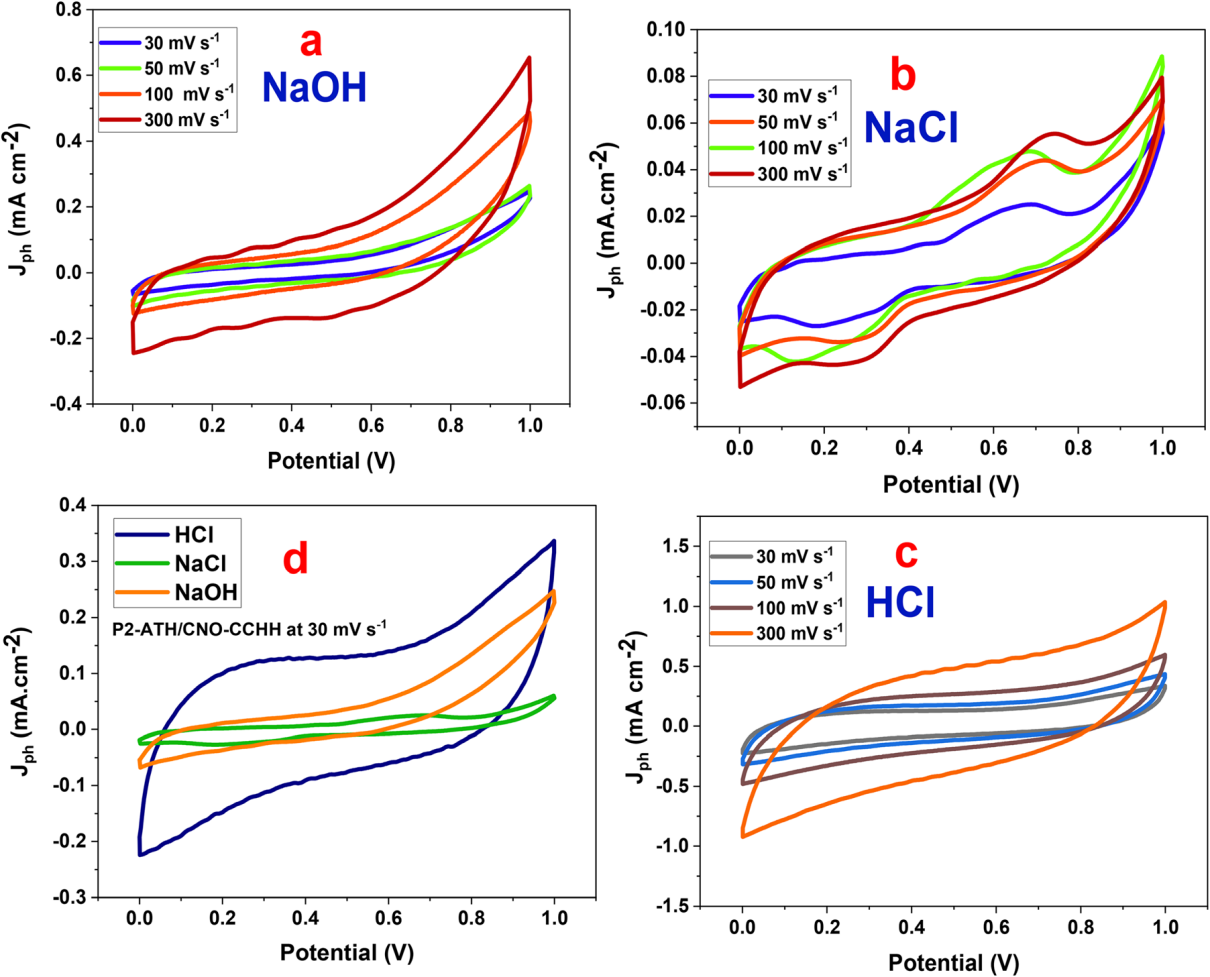
Cyclic voltammograms with various scan rates, P2-ATH//CNO-CCHH heterostructure in multiple electrolytes. A g^−1^. (a) NaOH, (b) NaCl, (c) HCl electolytes, (d) GCD in various electrolytes at 0.4 A g^−1^.

Fig. 8 displayed the CV test in three different electrolytes. This test was investigated over a potential window of 0.0–1.0 V and scan rates of 30–300 mV s^−1^. The CV profiles exhibit distinct electrochemical behaviors, highlighting the influence of ionic conductivity and electrolyte type on the charge storage mechanisms.

Generally, the current density and the enclosed area increase with scan rate across all electrolytes, reflecting typical capacitive behavior. At higher sweep rates, the potential is scanned more rapidly, leading to faster ion accumulation at the electrode surface and accelerating surface redox reactions. Consequently, the electrode stores and releases charge more quickly. Since more charge is cycled per unit time, the current density increases and the enclosed CV area expands.

The CV curves in NaOH (alkaline electrolyte) show a quasi-rectangular shape with slight distortions at higher potentials. Charge storage is primarily governed by EDLC, with minor contributions from pseudocapacitive effects associated with Ni(OH)_2_/CoOOH redox transitions.^46,47^ Although the applied current density increases with scan rate, the non-linear growth of the enclosed area reflects ion-diffusion limitations. The relatively large hydrated radius and sluggish diffusion of OH^−^ ions limit ion accessibility, resulting in moderate capacitance.

The CV curves in NaCl (neutral electrolyte) deviate further from ideal capacitive behavior, showing weaker redox peaks and smaller enclosed areas. The reduced current densities and peak broadening at higher scan rates indicate sluggish ion transport, increased polarization, and incomplete charge transfer. These effects are linked to the low ionic conductivity of Na^+^ and Cl^−^ and the absence of strongly participating redox species, which hinder efficient access to electroactive sites. Consequently, NaCl provides the lowest capacitance among the tested electrolytes.

The CV curves in HCl retain nearly symmetrical rectangular shapes, with the largest enclosed areas and the highest current densities. The profiles remain stable at elevated scan rates, confirming excellent reversibility and rate capability. This superior behavior is attributed to the small radius and high mobility of H^+^ ions, which enable quick diffusion, efficient penetration into electroactive sites, and rapid proton-coupled electron transfer (PCET). In addition, protonation-deprotonation of nitrogen- and sulfur-functional groups in P2-ATH, coupled with Co^2+^/Co^3+^ and Ni^2+^/Ni^3+^ redox transitions in CNO and CCHH, further enhances pseudocapacitance. These synergistic effects minimize internal resistance and enable efficient utilization of active sites even at high scan rates.^48^

A direct comparison at a fixed scan rate of 30 mV s^−1^ confirms these trends, as seen in Fig. 8(d). HCl delivers the largest enclosed area and current response, followed by NaOH, which shows moderate performance, while NaCl exhibits the weakest behavior. Notably, the electrochemical performance is not governed solely by proton mobility but is strongly influenced by the activation state of P2-ATH functional groups. In NaOH, the –NH_2_ and –SH groups remain predominantly deprotonated, leading to limited electronic conductivity and moderate capacitance, despite partial contributions from Ni(OH)_2_/CoOOH redox reactions. In NaCl, the absence of protonation, combined with low ionic conductivity and weak ion surface interactions, further suppresses redox activity, resulting in the lowest capacitance. In contrast, in HCl, protonation of –H_2_ to –NH_3_^+^ and polarization of sulfur sites markedly enhance polymer conductivity and activate proton-coupled electron transfer (PCET), thereby yielding superior capacitive behavior. Consequently, the inferior performance in NaOH and NaCl arises from both kinetic and thermodynamic limitations, whereas the outstanding performance in HCl is attributed to the synergistic interplay between rapid proton transport, effective functional group activation, and efficient utilization of redox-active sites within the porous hierarchical P2-ATH/CNO-CCHH architecture. These results underscore that electrolyte selection is decisive in optimizing charge storage, with acidic HCl offering the most favorable conditions for this heterostructure.

### 3.3 Galvanostatic charge/discharge (GCD)

Fig. S3 compares the GCC behavior of the pristine CNO-CCHH electrode and the P2-ATH/CNO-CCHH heterostructure in 0.5 M HCl. The CNO-CCHH electrode exhibits a discharge duration of approximately 75 s, indicating that charge storage is primarily governed by faradaic redox reactions associated with the Ni^2+^/Ni^+3^ and Co^2+^/Co^+3^ couples. The relatively short total charge/discharge time and noticeable IR drop suggest limited electrical conductivity, incomplete utilization of redox-active sites, and restricted proton diffusion within the oxide network.

After integration with P2-ATH, the total charge/discharge time of the P2-ATH/CNO-CCHH heterostructure increases markedly to approximately 177 s, representing more than a twofold enhancement in charge-storage capacity. This substantial increase confirms a strong synergistic effect between the two components. The CNO-CCHH phase serves as the primary faradaic charge-storage contributor due to its abundant redox-active metal centers, while the P2-ATH phase provides complementary pseudocapacitive and conductive functions. The conjugated polymer backbone facilitates rapid electron transport and bridges adjacent nanoneedles, thereby reducing charge-transfer resistance and improving charge propagation throughout the electrode. In addition, the nitrogen- and sulfur-containing functional groups in P2-ATH provide additional redox-active sites and promote proton-coupled electron transfer in acidic media, further enhancing the overall electrochemical performance.

**Fig. 9:**
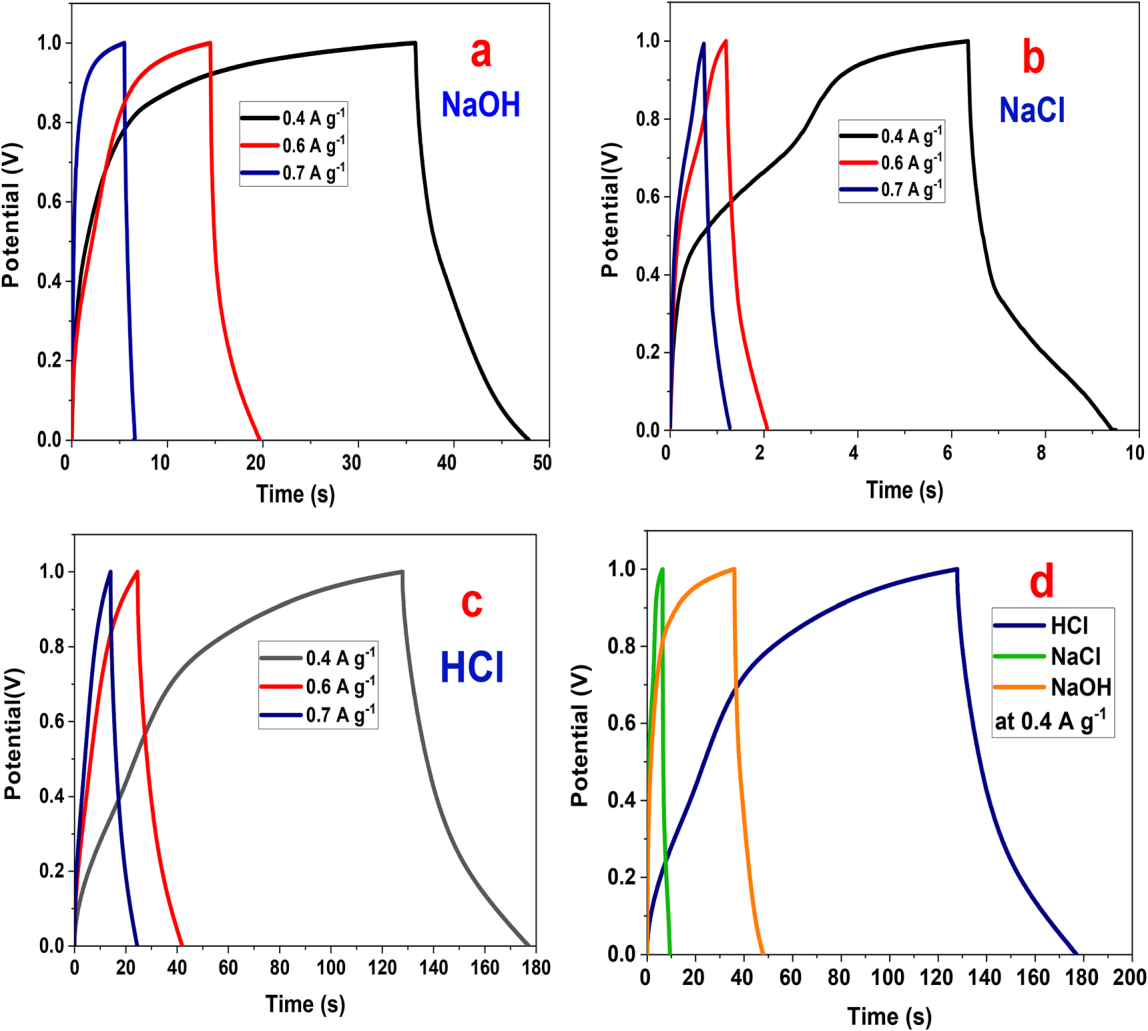
Galvanostatic charge/discharge of P2-ATH/CNO-CCHH electrode (a) NaOH, (b) NaCl, (c) HCl electrolytes, (d) GCD in various electrolytes at 0.4 A g^−1^.

The GCD analysis of the P2-ATH/CNO-CCHH heterostructure in HCl, NaOH, and NaCl electrolytes reveals pronounced differences in electrochemical. Fig. 9 shows that the electrode exhibits well-defined charge/discharge profiles across all electrolytes, but the discharge times and overall shapes vary significantly.

The charge/discharge time decreases with an increase in current density.^49,50^ The ions are afforded sufficient time to penetrate the porous heterostructure at lower current densities. This enables the fuller utilization of electroactive sites, resulting in longer discharge durations. In contrast, increasing current density restricts ionic access to the electrode interior, resulting in shorter charge/discharge cycles.^51,52^ This inverse relationship between current density and discharge duration is a typical characteristic of supercapacitive materials and confirms the mixed capacitive-pseudocapacitive nature of the P2-ATH/CNO-CCHH electrode.

In NaOH electrolyte, the discharge durations are moderate (50 s at 0.4 A g^−1^), accompanied by slightly larger IR drops than those in HCl. The profiles retain a quasi-triangular shape but with subtle deviations that reflect slower diffusion kinetics. The hydrated OH^−^ ions, larger and less mobile than protons, limit ionic transport and reduce the utilization of internal electroactive sites. Nevertheless, pseudocapacitive contributions from Ni(OH)_2_/CoOOH redox processes partially compensate for these limitations, resulting in moderate capacitance values.

NaCl exhibits the poorest performance, with discharge durations of only 10 s at 0.4 A g^−1^ and the largest IR drop among the three electrolytes. The distorted GCD profiles reflect significant polarization, sluggish ionic diffusion, and limited redox activity. The large hydrated radius of Na^+^ and the low ionic mobility of Na^+^ and Cl^−^ hinder efficient access to the electrode's active sites. At the same time, the absence of strongly participating redox species results in predominantly electric double-layer behavior with minimal pseudocapacitive enhancement.

A comparative evaluation at 0.4 A g^−1^ (Fig. 9(d)) highlights the substantial effect of electrolyte composition. In HCl, the GCD profiles exhibit the longest total charge/discharge time (177 s), nearly ideal triangular shapes, minimal IR drop, and strong charge/discharge symmetry. These features indicate low equivalent series resistance, fast charge-transfer kinetics, and high reversibility. The superior performance in HCl arises from the electrolyte's high ionic conductivity and the exceptional mobility of H^+^ ions, which have a small hydrated radius and can rapidly diffuse through the hierarchical porous nanoflake-nanoneedle architecture. Moreover, the acidic medium promotes protonation of the nitrogen- and sulfur-containing functional groups in the P2-ATH backbone, thereby enhancing polymer conductivity and facilitating proton-coupled electron transfer. This enables more efficient utilization of both the polymeric redox sites and the Ni/Co active centers.

To further elucidate electrolyte-dependent behavior, supplementary GCD measurements were performed in 0.5 M Na_2_SO_4_ and 0.5 M HClO_4_ (Fig. S4). In neutral Na_2_SO_4_, the P2-ATH/CNO-CCHH heterostructure exhibits a short total charge/discharge time of approximately 6.5 s, indicating limited charge-storage capability. This behavior arises from the large hydrated radii and low mobility of Na^+^ and SO_4_^2−^ ions, which hinder electrolyte diffusion through the hierarchical porous network and restrict access to internal electroactive sites. In contrast, in 0.5 M HClO_4,_ the electrode exhibits a longer total charge/discharge time of approximately 19.7 s, reflecting enhanced ionic conductivity and increased redox activity in the acidic medium. This moderate improvement results from the small hydrated radius and high mobility of H^+^ ions, which enable rapid ion diffusion and facilitate proton-coupled electron transfer (PCET) at the nitrogen- and sulfur-containing functional groups of P2-ATH, as well as at the Ni/Co redox centers. Despite this improvement, the performance in HClO_4_ remains markedly inferior to that in 0.5 M HCl, where the discharge duration reaches approximately 177 s (Fig. 9). This pronounced difference is governed by anion-specific effects on ionic transport, solution resistance, and interfacial kinetics. Although both acidic electrolytes supply highly mobile H^+^ ions, the smaller ionic radius and higher mobility of Cl^−^ confer superior bulk conductivity and lower ohmic polarization compared with the bulky ClO_4_^−^ anion. Moreover, chloride ions promote favorable interfacial adsorption, improved wettability, and deeper electrolyte penetration into the hierarchical nanoarchitecture, thereby enabling more effective activation of the nitrogen- and sulfur-containing functional groups in the P2-ATH backbone and the Ni/Co redox centers. Hence, the GCD results confirm that 0.5 M HCl is the most favorable electrolyte for the P2-ATH/CNO-CCHH heterostructure, as it provides high proton mobility, low internal resistance, superior electrolyte conductivity, efficient ion transport, and effective utilization of redox-active sites.

### 3.4 Specific capacitance and Ragone plot

The GCD technique can be used to evaluate the specific capacitance (*C*_sp_) of the P2-ATH/CNO-CCHH heterostructure using eqn (5) (ref. 53)

5 *C*_sp_(F g^−1^) = 4*I*·Δ*t/*Δ*V*·*m*

where *I* is the applied current density (A g^−1^), Δ*V* is the potential window (V), Δ*t* is the discharge time (s), and *m* is the mass of the active material on the electrode (g).

**Fig. 10:**
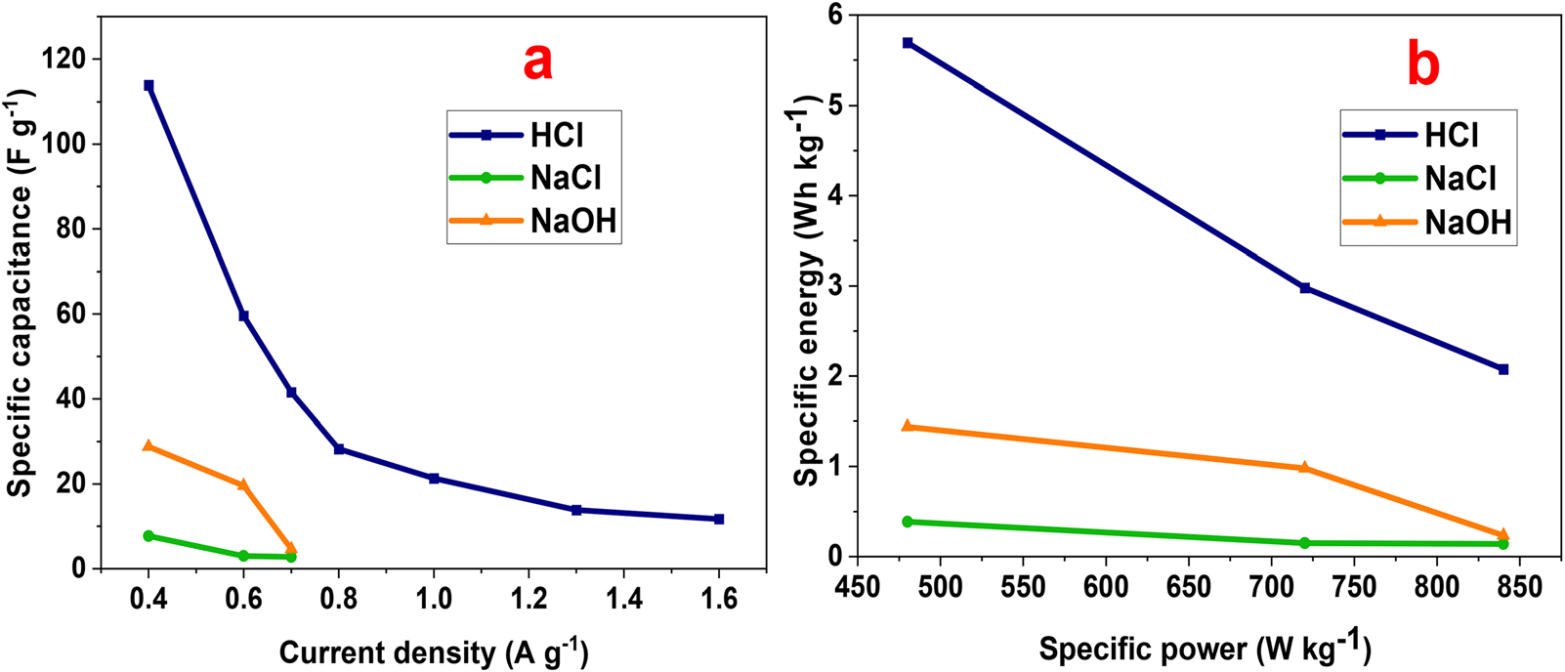
(a) Specific capacitance and (b) Ragone plot of the P2-ATH/CNO-CCHH electrode in different electrolytes.

Fig. 10(a) presents the dependence of specific capacitance on the current density. Specific capacitance declines with growing current density, a common feature in supercapacitor systems.^54^ At high current density, the reduced time available for ion diffusion limits access to the heterostructure's inner active sites, leading to incomplete redox reactions and lower charge storage efficiency. At 0.4 A g^−1^, the measured capacitances were 27.89, 7.73, and 113.87 F g^−1^ in NaOH, NaCl, and HCl electrolytes, respectively, underscoring the dominant influence of electrolyte composition.

The superior performance in HCl arises from its high ionic conductivity and the exceptional mobility of protons. Proton-coupled electron transfer, combined with protonation-deprotonation of nitrogen and sulfur sites in the P2-ATH backbone, enhances charge transport. Furthermore, Co^2+^/Co^3+^ and Ni^2+^/Ni^3+^ transitions in the CNO-CCHH phases provide additional pseudocapacitive contributions. These processes collectively enable efficient utilization of electroactive sites, consistent with the larger CV areas and longer discharge durations observed in HCl compared to neutral and alkaline media.

The structural characteristics of the heterostructure further reinforce this behavior. Integrating nanoneedles and nanoflakes into a 3D architecture maximizes surface area, accelerates electrolyte diffusion, and increases the density of electroactive sites. CNO-CCHH nanoneedles provide abundant redox-active centers and facilitate electron/ion transport, while the sheet-like P2-ATH polymer enhances conductivity and offers additional binding sites for interfacial reactions.^55^ In acidic environments, protonation of heteroatoms in P2-ATH further improves ionic conductivity, contributing to the high capacitance values observed.

The energy and power parameters offer key insights into the balance between energy density and power delivery, which are crucial for evaluating the electrode's applicability in practical supercapacitor devices. The energy-power characteristics of the electrode were assessed according to the following relations.

6 *E* (Wh kg^−1^) = 0.5 *C*_sp_ (Δ*V*)^2^

7 *P* (W kg^−1^) = *E*/Δ*t*

where *C*_sp_ is the specific capacitance (F g^−1^), Δ*t* is the discharge time (s), and Δ*V* is the potential window (V).

The results are illustrated in the Ragone plot (Fig. 10(b)). The calculated specific energies were 1.39, 0.39, and 5.69 Wh kg^−1^ for NaOH, NaCl, and HCl, respectively. The maximum specific power reached 479.7 W kg^−1^ in HCl. Notably, the electrode in HCl maintained energy densities above 4 Wh kg^−1^ even at high power densities, demonstrating brilliant rate capability and energy retention.

Taken together, these results emphasize that the P2-ATH/CNO-CCHH heterostructure achieves its best performance in acidic medium, where synergistic effects between proton transport, redox-active centers, and hierarchical nanoarchitecture maximize both capacitance and energy density.

### 3.5 Electrochemical stability

Fig. S5 illustrates the GCD cycling performance of the P2-ATH/CNO-CCHH heterostructure over 1000 consecutive cycles at a current density of 0.4 A g^−1^ in 0.5 M HCl. The electrode retained 96.8% of its initial capacitance after 1000 cycles. This corresponds to a capacitance loss of only 3.2%. The excellent retention demonstrates the robust stability of the heterostructure under repeated charge/discharge conditions.

The high cycling durability can be attributed to several factors. The synergistic interaction between the conducting polymer (P2-ATH) and the mixed metal oxide nanostructures (CNO and CCHH) ensures stable redox activity. The crystalline nature of the inorganic phases provides mechanical integrity and suppresses structural degradation. In addition, the hierarchical nanoneedle-nanoflake morphology accommodates volume changes during cycling while preserving the active surface area. The enhanced conductivity of the composite further minimizes resistive losses and facilitates rapid electron and ion transport during prolonged cycling. Collectively, these features enable the P2-ATH/CNO-CCHH heterostructure to exhibit excellent electrochemical stability, making it highly suitable for long-term energy storage applications.

### 3.6 EIS analysis

**Fig. 11:**
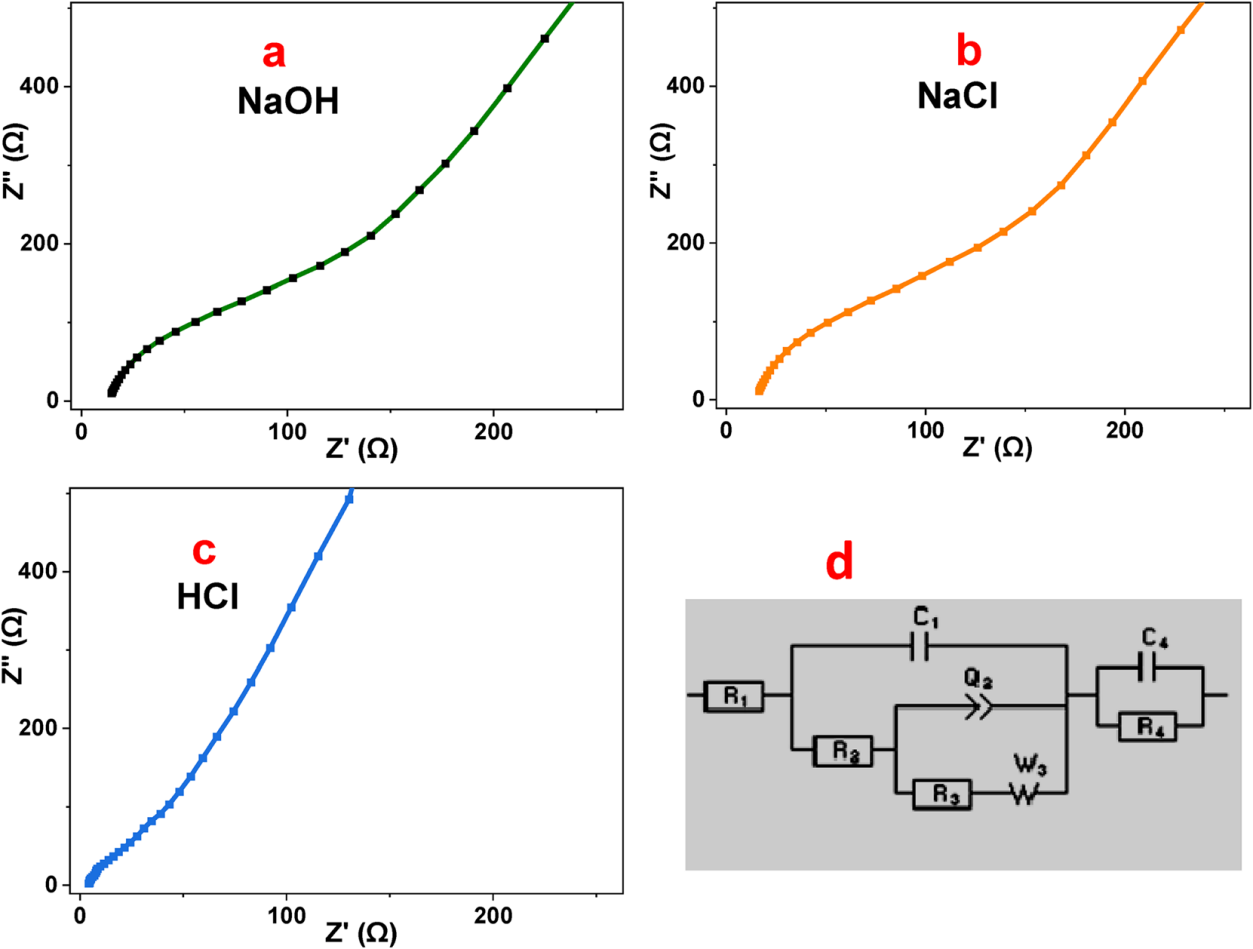
(a–c) EIS of P2-ATH/CNO-CCHH heterostructure in different electrolytes, and (d) the equivalent Rundle's circuit.

Electrochemical impedance spectroscopy (EIS) was employed to elucidate the charge-transfer kinetics and ion diffusion behavior of the P2-ATH/CNO-CCHH heterostructure at 0.5 M of NaOH, NaCl, and HCl electrolytes.^56^ The Nyquist plots presented in Fig. 11(a–c) illustrate the relationship between the real (Z′) and imaginary (Z″) components of impedance.^57^ The fitted data using EC-Lab software are represented by the equivalent Randles circuit shown in Fig. 11(d).

The high-frequency intercept on the real axis corresponds to the solution resistance (*R*_s_), which reflects the combined resistance of the electrolyte, electrode material, and electrical contacts.^58^ The semicircular region observed at intermediate frequencies is attributed to the charge-transfer resistance (*R*_ct_), representing the kinetics of faradaic reactions at the electrode–electrolyte interface. At low frequencies, the inclined or nearly vertical line is associated with Warburg impedance, which describes ion diffusion within the porous nanoflake-nanoneedle architecture.^59^ A smaller semicircle and a steeper low-frequency slope indicate faster charge-transfer processes and more efficient ion transport.

In alkaline (0.5 M NaOH) and neutral (0.5 M NaCl) electrolytes, the Nyquist plots exhibit relatively large semicircles, indicating significant interfacial resistance. The NaOH electrolyte shows *R*_s_ = 19.92 Ω and *R*_ct_ = 151 Ω, whereas NaCl exhibits *R*_s_ = 19.0 Ω and a higher *R*_ct_ of 185.5 Ω. These elevated resistance values reflect sluggish charge-transfer kinetics and limited faradaic activity. Although NaOH provides moderate ionic conductivity, the larger hydrated OH^−^ ions exhibit slower diffusion and weaker interaction with electroactive sites, resulting in only moderate capacitance (27.89 F g^−1^). In NaCl, the weak electrochemical participation of Cl^−^ ions and the limited mobility of Na^+^ further hinder ion transport and interfacial reactions, leading to the highest impedance and the lowest capacitance (7.73 F g^−1^).

In contrast, the acidic electrolyte (0.5 M HCl) displays a markedly improved impedance response, characterized by a very small semicircle and a nearly vertical line in the low-frequency region. The extracted parameters (*R*_s_ = 1.95 Ω and *R*_ct_ = 2.88 Ω) are substantially lower than those observed in NaOH and NaCl, indicating minimal ohmic losses and highly efficient charge-transfer processes.^60^ The rapid ion diffusion is attributed to the high mobility and small hydrated radius of H^+^ ions, which enable efficient penetration into the hierarchical pore network. Moreover, the acidic environment promotes protonation of the nitrogen- and sulfur-containing functional groups in the P2-ATH polymer backbone, significantly enhancing its electrical conductivity and facilitating proton-coupled electron transfer. The strong interfacial coupling between the conducting polymer and the redox-active CNO-CCHH phases reduces the energy barrier for faradaic reactions, leading to a pronounced decrease in *R*_ct_ (approximately 98% lower than that of NaCl). This synergistic interaction, combined with the interconnected three-dimensional architecture, establishes continuous pathways for efficient electron and ion transport.

These findings confirm that the superior behavior observed in HCl is directly responsible for the highest specific capacitance and improved energy storage performance of the P2-ATH/CNO-CCHH heterostructure.

Finally, Table 1 presents a comparative evaluation of the P2-ATH/CNO-CCHH heterostructure against recently reported conducting-polymer-based hybrid electrodes.^61–65^ When benchmarked against systems such as NiS-P2ATP/NiO core–shell structures, Fe_2_O_3_/poly(2-aminothiophenol) dumbbell-like architectures, nickel cobalt oxide/polyvinyl alcohol composites, poly(2-chlorobenzenamine)/AgCl nanospheres, and silver nanowire/PDOPEQ hybrids, the present electrode exhibits superior capacitance and rate-capability metrics, confirming its competitiveness among state-of-the-art materials.

**Table 1:** Comparative electrochemical performance of the P2-ATH/CNO-CCHH electrode and reported polymer-based hybrid supercapacitor materials

Material	Morphology	Current density (A g^−1^)	Electrolyte	Specific capacitance	Stability	Current collector	Ref.
NiS-P2ATP/NiO	Core–shell nanocomposite	0.2 A g^−1^	1 M H_2_SO_4_	59.8 F g^−1^	94% after 5000 cycle	Au sheet	61
Fe_2_O_3_/poly-2-aminothiophenol	Dumbbell-like shape nanocomposite	0.2 A g^−1^	1.0 M NaOH	44.5 F g^−1^, 9 Wh kg^− 1^	98.9% after 200 cycle	Au sheet	62
Nickel cobalt oxide-polyvinyl alcohol	Nanostructure composite films	0.2 A g^−1^	1 M Na_2_SO_4_	50 F g^−1^, 18.9 Wh kg^−1^	90% after 1000 cycle	Ni foam	63
poly(2-chlorobenzenamin)/AgCl	Nanospherical composite	0.2 A g^−1^	1 M H_2_SO_4_	86 F g^−1^	98.3% after 1000 cycles	Au sheet	64
Silver nanowire/PDOPEQ	Nanocomposite	0.1 A g^−1^	1 M LiClO4	61.5 F g^−1^	82.5% after 1000 cycles	Au sheet	65
P2-ATH/CNO-CCHH	Nanoflake–nanoneedle heterostructures	0.4 A g^−1^	0.5 HCl	113.87 F g^−1^, 479.7 W kg^−1^	96.8% after 1000 cycles	Au sheet	This work

Beyond its electrochemical advantages, the large-scale practical application of the P2-ATH/CNO-CCHH heterostructure is supported by the scalability and economic efficiency of its fabrication methods. Both hydrothermal synthesis and *in situ* oxidative polymerization are low-temperature, solution-based processes that can be readily adapted to industrial batch or continuous-flow production without the need for high-vacuum or high-temperature equipment. The use of earth-abundant Co/Ni salts and cost-effective polymer precursors further reduces raw-material expenses and enables high-volume manufacturing. Together, these factors underscore the material's strong potential for practical deployment in next-generation supercapacitors.

## 4 Conclusion

A P2-ATH/CNO-CCHH electrode was prepared by hydrothermal growth of Co–Ni carbonate/oxide nanoneedles followed by *in situ* oxidative polymerization of poly(2-aminothiophenol), yielding a hierarchical nanoflake-nanoneedle network. Structural and morphological analyses confirmed intimate interfacial contact, a large accessible surface area, and continuous ion and electron transport pathways. Electrochemical testing by CV and GCD in NaOH, NaCl, and 0.5 M HCl showed a clear electrolyte dependence, with the acidic medium giving the best response: a specific capacitance of 113.87 F g^−1^ at 0.4 A g^−1^, an energy density of 5.69 Wh kg^−1^, and a power density of 479.7 W kg^−1^, together with robust cycling stability. The improved performance in HCl is attributed to the combined action of the conductive P2-ATH shell and the redox-active Co–Ni phase, facilitated by rapid proton transport, which reduces charge-transfer resistance and enhances active-site utilization. These results identify the P2-ATH/CNO-CCHH architecture as a highly promising electrode design for achieving high-performance energy-storage systems.

## Conflicts of interest

The authors have no conflicts of interest.

## Supplementary Material

RA-016-D5RA09024E-s001

## Data Availability

The datasets generated and/or analysed during the current study are included in this published article. Further data are available from the corresponding author upon reasonable request. Supplementary information (SI): additional experimental details, a schematic of the P2‑ATH/CNO‑CCHH heterostructure preparation (Fig. S1), and FTIR band assignments for the P2‑ATH/CNO‑CCHH heterostructure (Table S1). See DOI: https://doi.org/10.1039/d5ra09024e.
