# Supplementary material for: Hierarchical poly(2-aminothiophenol)/Co–Ni heterostructures with nanoflake–nanoneedle architecture for high-performance supercapacitors
Source: RSC Adv. 2026 May 8;16(26):24256–70. doi: 10.1039/d5ra09024e (PMC13154781; doi:10.1039/d5ra09024e)
Supplement: RA-016-D5RA09024E-s001 [file RA-016-D5RA09024E-s001.pdf]

Supplementary Information (SI) for RSC Advances.
This journal is © The Royal Society of Chemistry 2026

Hierarchical Poly(2-aminothiophenol)/Co-Ni Heterostructures with Nanoflake-Nanoneedle Architecture for High-Performance Supercapacitors

Aya Fathy [1], Ashour M. Ahmed [2], M. A. Basyooni-M. Kabatas [3,4*], Mamduh J. Aljaafreh [2], Mohamed Shaban [5], Hany Hamdy [1], Mohamed Rabia [6]

[1] Nanophotonics and Applications Lab, Physics Department, Faculty of Science, Beni-Suef University, Beni-Suef 62514, Egypt

[2] Physics Department, College of Science, Imam Mohammad Ibn Saud Islamic University (IMSIU), Riyadh 11623, Saudi Arabia

[3] Department of Precision and Microsystems Engineering, Delft University of Technology, Mekelweg 2, 2628 CD Delft, The Netherlands

[4] Institute of Nanotechnology (INT), Karlsruhe Institute of Technology (KIT), Kaiserstraße 12, 76131 Karlsruhe, Germany

[5] Physics Department, Faculty of Science, Islamic University of Madinah, Madinah, Saudi Arabia

[6] Nanomaterials Science Research Laboratory, Chemistry Department, Faculty of Science, Beni-Suef University, Beni-Suef, Egypt

* Corresponding author: M. A. Basyooni-M. Kabatas (m.kabatas@tudelft.nl or m.kabatas@kit.edu)

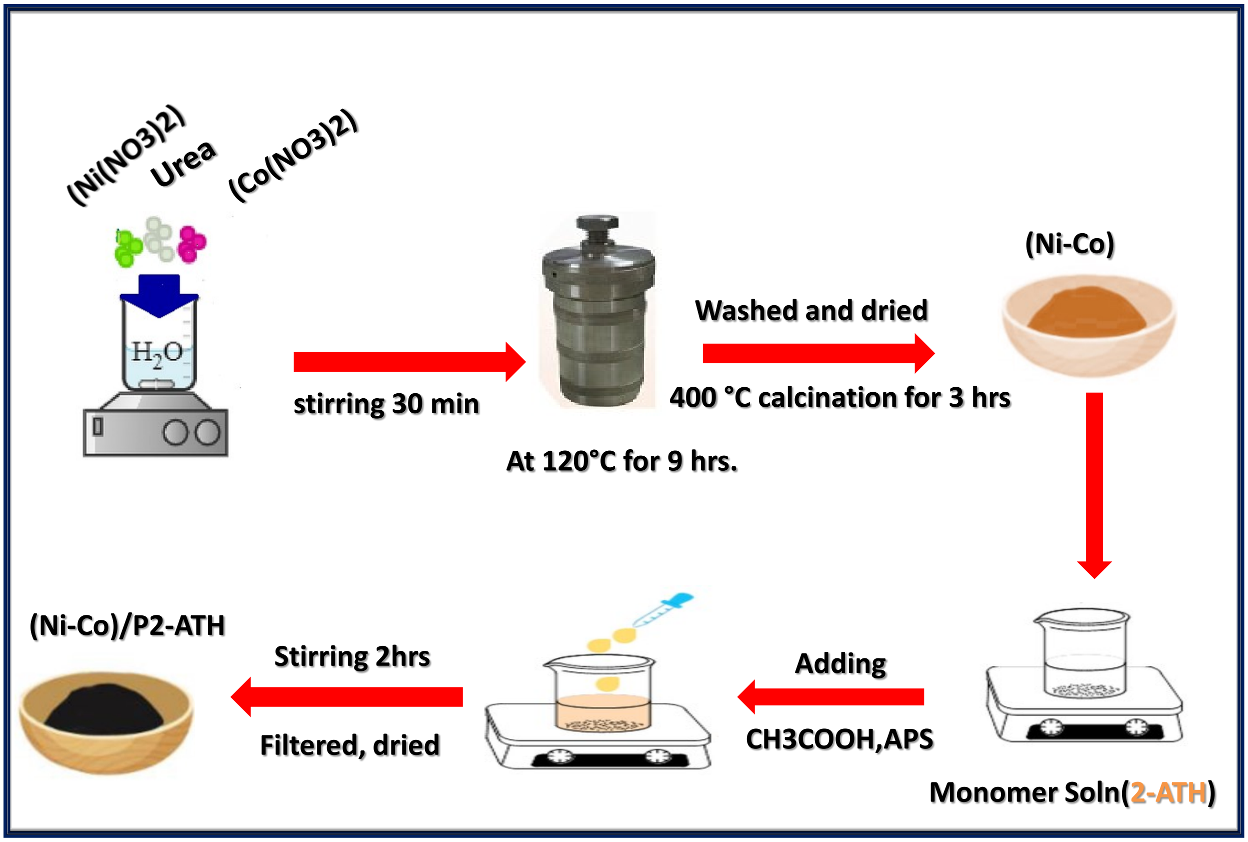

Figure S1. The schematic diagram for the preparation of P2-ATH/CNO-CCHH heterostructure

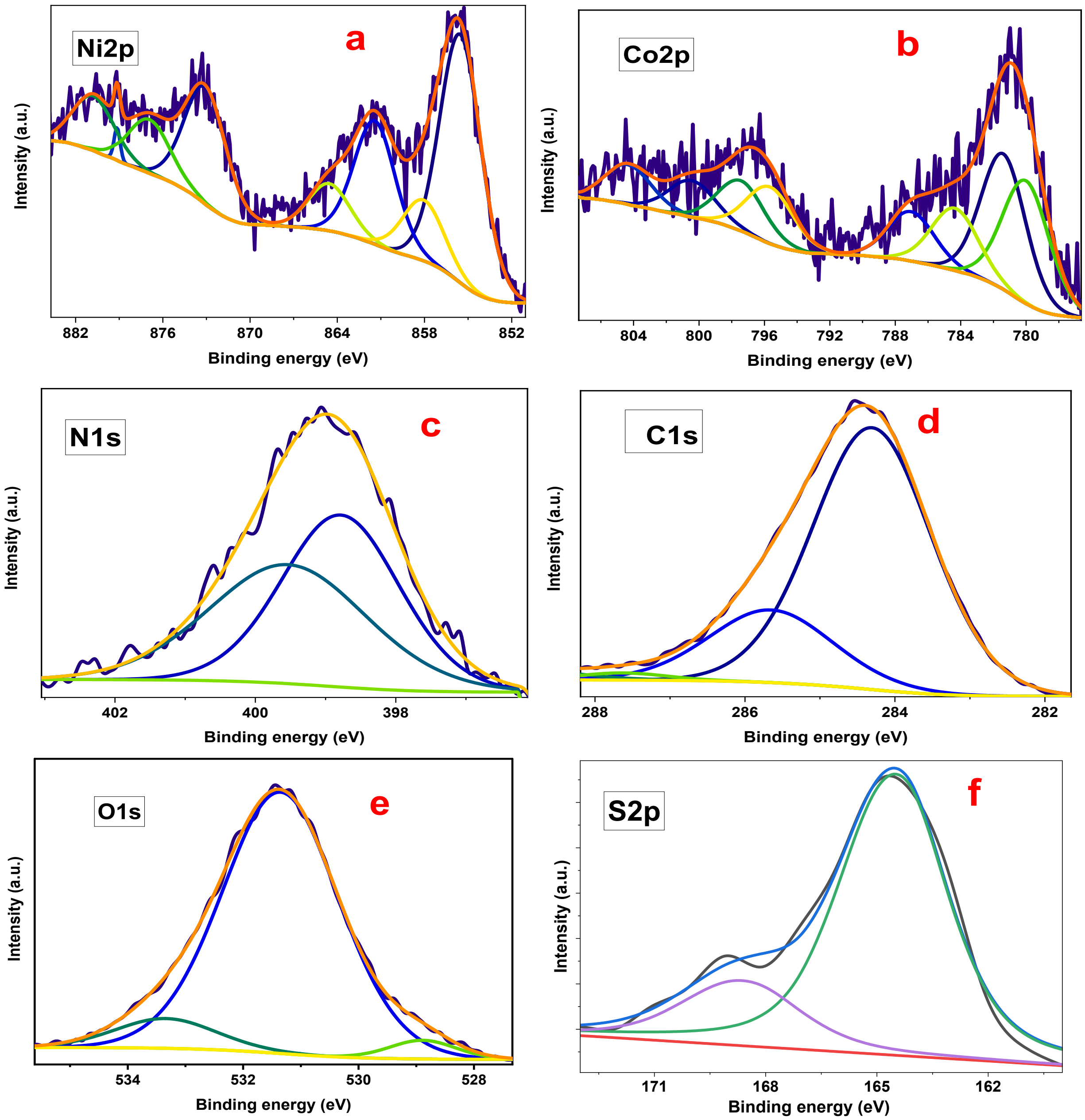

Figure S2. High-resolution XPS analysis of P2-ATH/CNO-CCHH heterostructure; (a) Ni2p, (b) Co2p, (c) N1s, (d) C1s, (e) O1s, and (f) S2p

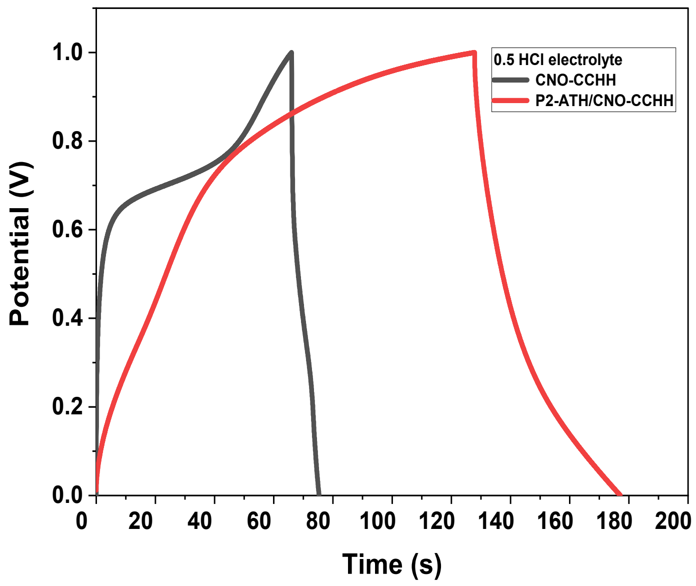

Figure S3. GCD profiles of CNO-CCHH and P2-ATH/CNO-CCHH electrodes in 0.5 HCl

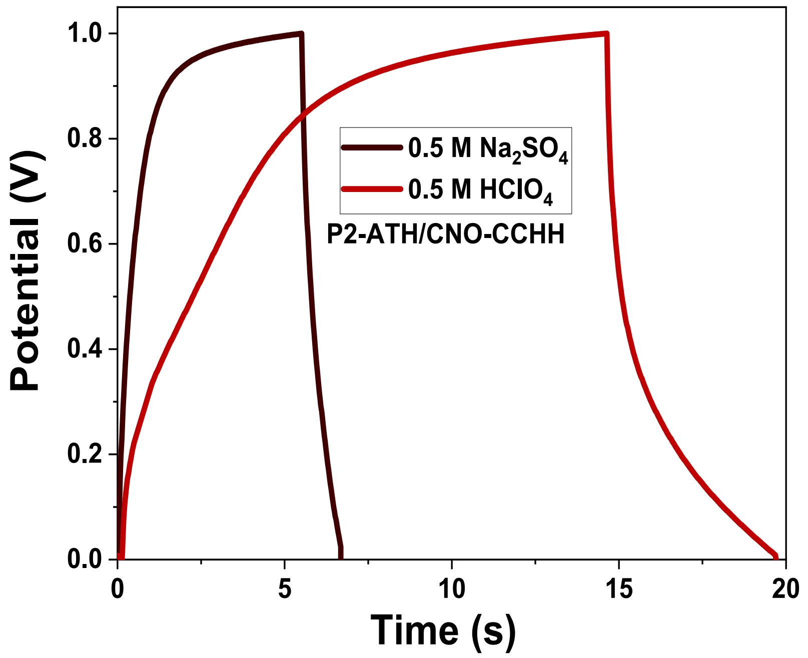

Figure S4. GCD profiles of P2-ATH/CNO-CCHH electrodes in 0.5 M Na_2SO_4 and 0.5 M $HClO_4$

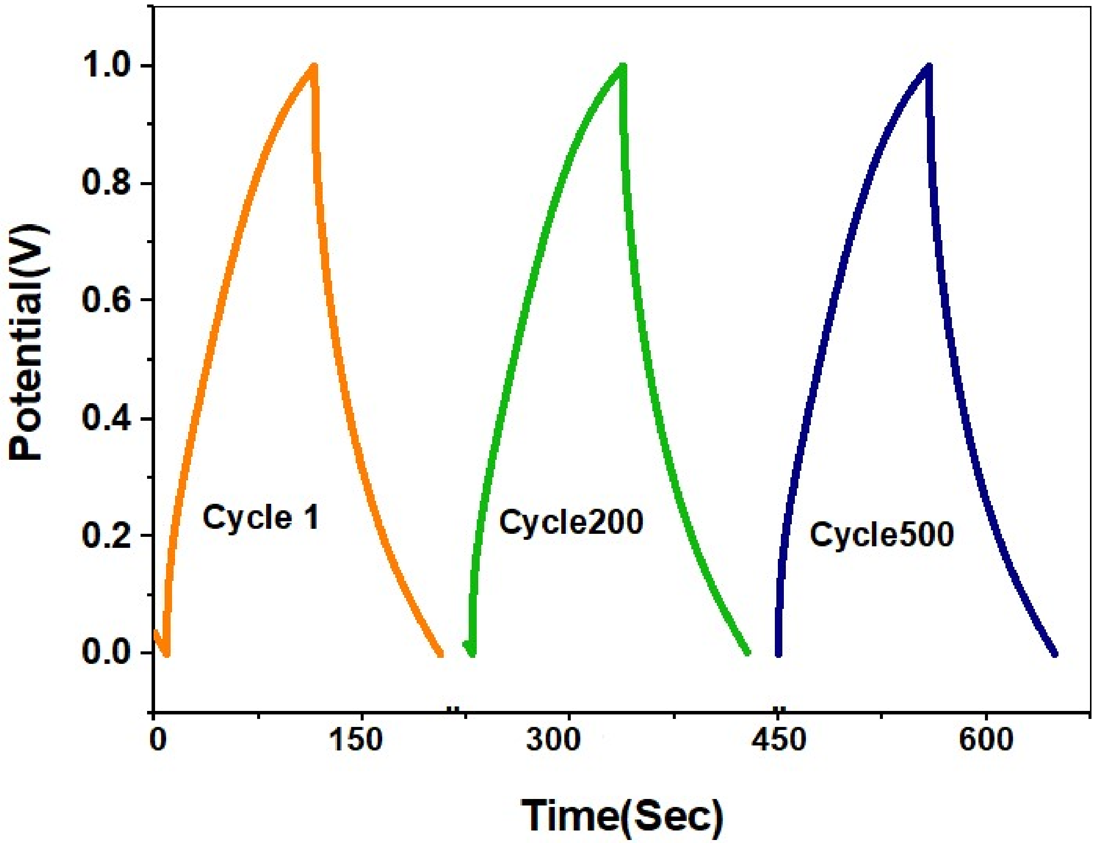

Figure S5. The stability performance of P2-ATH/CNO-CCHH heterostructure in 0.5 M HCl for 1000 cycles

Table S1. The FTIR analysis of the P2-ATH/CNO-CCHH heterostructure

**Band Position (cm^{-1})**	**Assignment**	**Ref**
3756.5 - 3692.6	Broadband for stretching vibration of hydroxyl groups (OH) linking with Co and Ni atoms.	[S1]
3354 - 3438.1	N-H, S-H stretching of the primary amine confirms the amino group	[S2, S3]
3054.4	C-H stretching vibration of the various alkane groups	[S4]
1470.1	C=C vibration in the benzenoid ring	[S5]

1376.5	C-N stretching vibrations	[S6]
1124.8	Ni-O stretching vibrations	[S7]
1040.2	C-H in-plane bending vibration	[S8]
962.2	C-S group band	[S9]
825.2	Out-of-plane C-H deformation showing ortho disubstitution in benzene ring	[S2]
752.2	Indicating specific chemical interactions within the polymer structure	[S10]
634.4	Carbonate group (CO_3^{2-})	[S11, S12]
539.2	Bonds of Ni-O and Co-O with vibrations overlapping	[S13]
451.5	Co-OH stretching	[S12]

Reference:

S1. Wang, J., et al., Quantitative determination of titanium lattice defects and solid-state reaction mechanism in iron-doped TiO2 photocatalysts. 2001. **105**(40): p. 9692-9698.

S2. Rabia, M. and M.A.J.J.o.E.M. Alnuwaiser, Fabrication of a Photocathode Based on Silver Iodide/Poly-2-Aminothiophenol with High Optical Absorbance Related to Intercalated Iodide Ions for Hydrogen Generation from Wastewater Without a Sacrificial Agent. 2024: p. 1-13.

S3. Nabid, M.R., et al., Preparation and application of poly (2-amino thiophenol)/MWCNTs nanocomposite for adsorption and separation of cadmium and lead ions via solid phase extraction. 2012. **203**: p. 93-100.

S4. Rana, S.B., et al., Influence of surface modification by 2-aminothiophenol on optoelectronics properties of ZnO nanoparticles. 2014. **9**(9): p. 877-891.

S5. Mosa, J., et al., Synthesis of poly (phenylene oxide)-based fluoro-tin-oxide/ZrO2 nanoelectrode arrays by hybrid organic/inorganic approach. 2011. **56**(20): p. 7155-7162.

S6. Sayyah, S., M. Shaban, and M.J.A.i.P.T. Rabia, Electropolymerization of m-toluidin on platinum electrode from aqueous acidic solution and character of the obtained polymer. 2018. **37**(1): p. 126-136.

S7. ur Rehman, M.N., et al., Facile synthesis and characterization of conducting polymer-metal oxide based core-shell PANI-Pr2O–NiO–Co3O4 nanocomposite: As electrode material for supercapacitor. 2021. **47**(13): p. 18497-18509.

S8. Boomi, P., et al., Improved conductivity and antibacterial activity of poly (2-aminothiophenol)-silver nanocomposite against human pathogens. 2018. **178**: p. 323-329.

S9. Kabel, K.I., et al., Assessment of corrosion inhibitive behavior of 2-aminothiophenol derivatives on carbon steel in 1 M HCl. 2015. **23**: p. 57-66.

S10. Rabia, M., E. Aldosari, and A.A.A.J.J.o.M.S.M.i.E. Abdelazeez, An advanced optoelectronic apparatus utilizing poly (2-amino thiophenol) adorned with a needle-shaped MnS-MnO2 nanocomposite. 2024. **35**(6): p. 377.

S11. Rahimi-Nasrabadi, M., et al., Cobalt carbonate and cobalt oxide nanoparticles synthesis, characterization and supercapacitive evaluation. Journal of Materials Science: Materials in Electronics 2017. **28**(2): p. 1877-1888.

S12. Guellati, O., et al., Electrochemical measurements of 1D/2D/3DNi-Co bi-phase mesoporous nanohybrids synthesized using free-template hydrothermal method. 2018. **275**: p. 155-171.

S13. Isari, A.A., et al., N, Cu co-doped TiO2@ functionalized SWCNT photocatalyst coupled with ultrasound and visible-light: an effective sono-photocatalysis process for pharmaceutical wastewaters treatment. 2020. **392**: p. 123685.
